# Left Atrial Appendage Occlusion Device Infection: A Rare Case Report and Literature Review

**DOI:** 10.7759/cureus.116075

**Published:** 2026-09-10

**Authors:** Abhishek Vadher, Rakesh Devireddy, Kesava Manikanta Achuta, Adam Chornoby, Ashok Kondur

**Affiliations:** 1 Internal Medicine, Garden City Hospital, Michigan State University, Garden City, USA; 2 Cardiology, Garden City Hospital, Michigan State University, Garden City, USA

**Keywords:** amplatzer amulet device, cardiac device-related infection, “infective endocarditis”, left atrial appendage occlusion, staph aures, watchman device

## Abstract

An 84-year-old male patient with a history of paroxysmal atrial fibrillation, status post dual-chamber permanent pacemaker and Watchman device implantation two weeks prior to presentation, came to the hospital with an acute onset of fevers, chills, and rigors. Blood cultures were positive for coagulase-negative *Staphylococcus* in two out of two bottles. Transthoracic echocardiography (TTE) was negative, and TEE showed a mobile echodensity measuring 1.4 × 1.2 cm attached to the Watchman device, consistent with a vegetation due to infective endocarditis (IE). He underwent surgical removal of the Watchman device along with explantation of the right atrial and ventricular pacemaker leads. He was discharged on oral cefuroxime and intravenous vancomycin for a total of six weeks.

We conducted a literature search of PubMed, Embase, Cochrane, Scopus, Web of Science, and Google Scholar. We found 14 cases of infected Left Atrial Appendage Occlusion (LAAO) devices. The Watchman device was the most commonly used, in 10 of 14 patients; the Amulet device was used in three patients, and one patient had an AtriClip. The diagnostic modality of choice was transesophageal echocardiography, which was performed in all cases. FDG PET/CT was performed in one case to determine FDG uptake and infection of the LAAO device. The most common organisms identified from blood cultures were gram-positive organisms, reported in 12 patients (85.7%). The predominant organism was *Staphylococcus aureus*, with six patients having methicillin-sensitive *Staphylococcus aureus* (MSSA) and three patients having methicillin-resistant *Staphylococcus aureus* (MRSA). Three patients had coagulase-negative *Staphylococcus*, including one patient in whom the organism was specifically identified as *Staphylococcus epidermidis*. Patients with MRSA infection were treated with vancomycin in one patient and ceftaroline with daptomycin in the other two patients. Patients with MSSA were treated with nafcillin, cefazolin with or without oral rifampicin, or flucloxacillin. Pseudomonas infection was treated with amikacin, cefotaxime, and ciprofloxacin. Mortality was noted in 4 of 14 patients (28.57%), and three patients died during hospitalization.

## Introduction

Atrial fibrillation is a very common arrhythmia worldwide. As per the Global Burden of Disease Study 2019, more than 55 million individuals have lived with atrial fibrillation [1]. One of the most important aspects of managing atrial fibrillation is stroke prevention through the administration of anticoagulant medications. The risk of thromboembolism is assessed using the CHA₂DS₂-VASc score [2]. However, a significant number of patients may not be good candidates for long-term anticoagulation due to a high risk of GI bleeding, medication noncompliance, or multiple falls. In atrial fibrillation, the predominant site of thrombus formation is the left atrial appendage. Left atrial appendage closure has emerged as an alternative management strategy to anticoagulant medications to reduce the risk of cardioembolic stroke in these patients. Several trials have demonstrated that Left Atrial Appendage Occlusion (LAAO) devices are non-inferior to oral anticoagulation for the prevention of thromboembolic outcomes in selected patients [3].

LAAO devices can be used as an alternative or supplemental strategy to anticoagulant medications in patients with atrial fibrillation [4]; however, there are some complications associated with LAAO devices, including device-related thrombus, peri-device leak, and device-related infections [5]. Over the past several years, percutaneous closure of the LAA has commonly been performed using either the Watchman device (Boston Scientific, Marlborough, MA, USA) or the Amplatzer Amulet device (Abbott, Minneapolis, MN, USA) [6]. Placement of any intracardiac foreign material carries a risk of infection. There is no study that reports the lifetime risk of infection after LAAO device placement. Olsen T et al. [7] reported that the lifetime risk of infection after pacemaker implantation is 1.19%, that for implantable cardioverter-defibrillators is 1.91%, that for cardiac resynchronization therapy pacemakers is 2.18%, and that for cardiac resynchronization therapy defibrillators (CRT-D) is 3.35%. The published literature on LAAO device infection remains limited, consisting predominantly of individual case reports and small case series, with substantial variation in clinical presentation, causative organisms, diagnostic evaluation, and management strategies [6]. Consequently, the clinical characteristics and optimal management of these infections remain incompletely defined.

In this study, we report the case of an 84-year-old man with a coagulase-negative *Staphylococcus* infection of the Watchman device, prompting surgical removal of the device. We also present a systematic review of the published literature to characterize reported LAAO device infections, summarize their clinical and microbiological features, and describe the diagnostic and management approaches used in these cases. By combining a detailed case report with a systematic review of the available evidence, this study aims to provide a more comprehensive description of this rare complication and identify areas requiring further investigation.

## Case presentation

An 84-year-old male with a history of paroxysmal atrial fibrillation, status post recent Watchman device placement 14 days prior to presentation, chronic diastolic heart failure, sick sinus syndrome with placement of a dual-chamber pacemaker, and chronic obstructive pulmonary disease presented with an acute onset of fever (101.4°F), chills, and rigors for seven days. About five days prior to the current presentation, the patient presented to the ER with similar complaints. Blood cultures and routine blood work were obtained, and he was offered observation, but he decided to follow up with his PCP and declined observation in the hospital. Subsequently, he returned to the ER with no improvement in his symptoms.

On initial examination, he was hemodynamically stable and had no signs of decompensated heart failure. The blood cultures from the previous ER visit revealed coagulase-negative *Staphylococcus* in two out of two bottles. The infectious disease team was consulted and believed that the cultures were probable contaminants and recommended continuing vancomycin pending repeat blood cultures. Transthoracic echocardiography (TEE) was normal and did not show any vegetation on the Watchman device or pacemaker leads. Repeat blood cultures revealed growth of *Staphylococcus epidermidis* in two out of four bottles. It was decided to proceed with transesophageal echocardiography per the recommendation of the Cardiology team. Transesophageal echocardiography demonstrated a mobile echodensity measuring 1.4 × 1.2 cm attached to the Watchman device, consistent with a vegetation due to infective endocarditis (IE) (Figure 1). The pacemaker leads did not show any evidence of vegetations.

**Figure 1:**
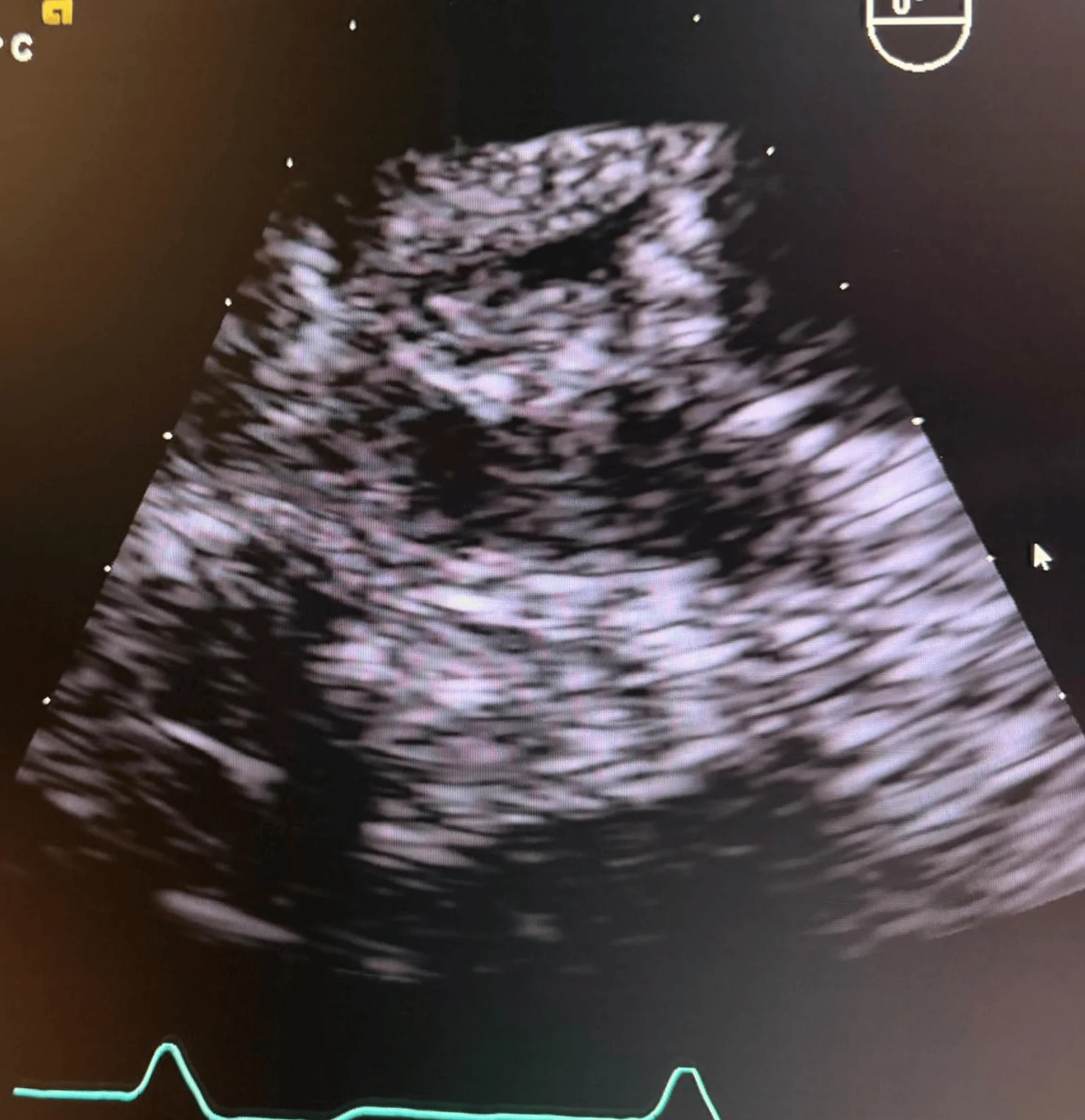
Transesophageal echocardiographic image at a biplane angle of 0° showing a mobile echodensity measuring 1.4 × 1.2 cm, which appears to be attached to the Watchman left atrial appendage occlusion device.

He was transferred to another facility for removal of the Watchman device by a cardiothoracic surgeon. Electrophysiology was consulted and recommended Watchman device extraction with a Maze procedure for atrial fibrillation. The right ventricular and right atrial pacemaker leads were also removed, along with the pacemaker generator in the right subclavian region. Although TEE did not demonstrate a definite vegetation involving the pacemaker leads, complete extraction of the pacemaker system was performed because of clinical concern for cardiac implantable electronic device (CIED) infection in the setting of endocarditis. A right ventricular epicardial lead was inserted for cardiac pacing. The patient was transferred to the cardiac ICU after the procedure and was eventually discharged with a total of six weeks of oral cefuroxime and intravenous vancomycin.

Two months later, the patient was readmitted to the hospital with severe hypoxia secondary to multifocal pneumonia and bilateral exudative pleural effusions, likely due to aspiration pneumonia. Broad-spectrum antibiotics were started, but the patient eventually decided to limit his care to palliation and passed away.

## Discussion

We searched the databases PubMed, Embase, Scopus, Cochrane, Google Scholar, and Web of Science. We used the keywords “left atrial appendage closure,” “left atrial appendage occlusion,” “LAAO,” “LAAC,” “Watchman,” “Amulet,” “infection,” “endocarditis,” “sepsis,” and “abscess.” The systematic review was conducted following PRISMA guidelines. We searched articles from inception to December 2025. Two authors (AV, RD) reviewed the literature independently. Exclusion criteria consisted of irrelevant topics, wrong populations, and wrong interventions/devices. Any discrepancy between the authors was resolved by the other authors (KMA, AC). The Preferred Reporting Items for Systematic Reviews and Meta-Analyses (PRISMA) flowchart is shown in Figure 2. Since the number of articles obtained after screening was limited (n=14), we included all the articles after full-text screening without conducting a formal quality/risk-of-bias assessment. The following variables were extracted for analysis: country, year, age, sex, diagnosis prior to the procedure, organism, LAA device, likely source of infection, clinical manifestation, time between the procedure and development of IE, medical therapy, surgical removal, vegetation size and location, embolization, cardiovascular manifestations, outcome, and follow-up.

**Figure 2:**
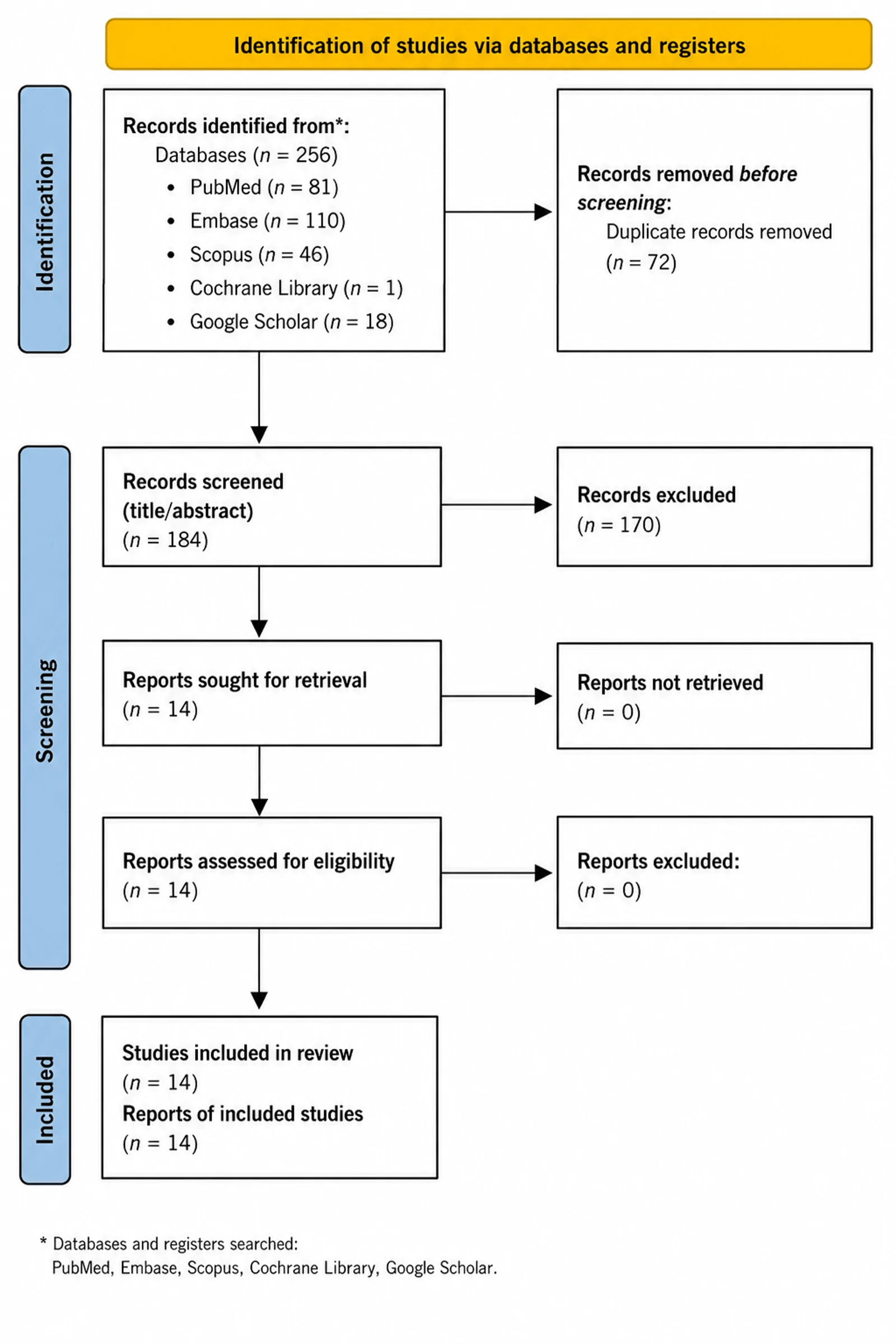
PRISMA flow diagram. PRISMA: Preferred Reporting Items for Systematic Reviews and Meta-Analyses.

Our study included a total of 15 patients (14 patients identified through the literature search and our patient). Baseline characteristics of the patients are documented in Table 1. The mean age of the patients was 73.4 years. Nine of 15 patients (60%) were male. Eleven cases were reported in the United States, two in Germany, one in France, and one in Spain. The first documented case was reported by Khumri TM et al. in the United States in 2008 [2] and involved an infected Watchman device. Six patients had paroxysmal atrial fibrillation, two patients had permanent atrial fibrillation, and 4 had persistent atrial fibrillation; the type was not mentioned in 3 patients. Two patients had dual-chamber pacemakers, 1 patient had a CRT-D, and one patient had a bioprosthetic aortic valve replacement. The Watchman device was the most commonly used, in 11 of 15 patients; the Amulet device was used in three patients, and one patient had an AtriClip. The size of the Watchman device was mentioned in three cases, with sizes of 21 mm, 24 mm, and 31 mm. The sizes of the Amulet devices were not mentioned. The size of the AtriClip was 50 mm. Preprocedural antibiotic prophylaxis was mentioned in only three cases; two patients (including our patient) did not receive intraoperative antibiotic prophylaxis. One patient received IV cefazolin during the procedure.

**Table 1:** Baseline characteristics of the patients. AF: Atrial Fibrillation; CAD: Coronary Artery Disease; CHA₂DS₂-VASc: Congestive Heart Failure, Hypertension, Age ≥75 Years, Diabetes Mellitus, Stroke/Transient Ischemic Attack/Thromboembolism, Vascular Disease, Age 65-74 Years, Sex Category; CRT-D: Cardiac Resynchronization Therapy Defibrillator; DES: Drug-Eluting Stent; GI: Gastrointestinal; HAS-BLED: Hypertension, Abnormal Renal/Liver Function, Stroke, Bleeding History or Predisposition, Labile International Normalized Ratio, Elderly, Drugs/Alcohol Concomitantly; LAAO: Left Atrial Appendage Occlusion; mm: Millimeter.

Study / Case	Country	Year	Age (years)	Sex	Diagnosis prior to procedure	LAAO Device	Clinical manifestations	Cardiovascular manifestations	Periprocedural antibiotics
Khumri TM et al. [8]	United States	2007	75	Female	Paroxysmal atrial fibrillation	24 mm Watchman	Sepsis, persistent bacteremia, chest pain, and lightheadedness	Not mentioned	None
Boukobza M et al. [9]	France	2017	83	Male	Ischemic cardiomyopathy with permanent atrial fibrillation	Amplatzer Amulet	Sepsis, subarachnoid hemorrhage, fever, and confusion	Systolic aortic and mitral murmurs	Not mentioned
Jensen J et al. [10]	United States	2020	74	Male	Persistent atrial fibrillation, coronary artery disease with drug-eluting stent	Watchman	Sepsis	Not mentioned	Not mentioned
Madanat L et al. [11]	United States	2021	74	Male	Persistent atrial fibrillation, CHA₂DS₂-VASc score of 4, HAS-BLED score of 3, prostate cancer status post radiation therapy, and metastatic melanoma on intravenous pembrolizumab	Watchman	Sepsis, persistent bacteremia, fatigue, myalgias, fever, and cough	Atrial fibrillation with rapid ventricular response	Not mentioned
von Roeder M et al. [12]	Germany	2020	54	Female	Diabetes, heart failure with reduced ejection fraction, morbid obesity (BMI 51 kg/m²), coronary artery disease, persistent atrial fibrillation, and CRT-D	Amplatzer Amulet	Bacteremia, fever, and chills	Not mentioned	Not mentioned
Al-Terki H et al. [6]	Germany	2022	83	Female	Permanent atrial fibrillation, hypertension, pulmonary hypertension, and severe tricuspid regurgitation	Amplatzer Amulet	Fever, chills, fatigue, and decreased alertness	Not mentioned	Not mentioned
Lazarus JJ et al. [13]	United States	2013	50	Male	Severe aortic stenosis, paroxysmal atrial fibrillation, hypertension, end-stage renal disease, HAS-BLED score of 4, and bioprosthetic aortic valve replacement	50 mm AtriClip	Fever, chills, headache, myalgia, and non-exertional sharp chest pain	Systolic murmur at the left lower sternal border	Not mentioned
Joury A et al. [14]	United States	2020	70	Male	Persistent atrial fibrillation, pancytopenia due to cryptogenic liver cirrhosis, recurrent urologic bleeding due to hemorrhagic cystitis, and type 2 diabetes mellitus	31 mm Watchman	Malaise, fatigue, an unwitnessed mechanical fall, and right foot pain	Not mentioned	Not mentioned
Jiménez Martínez C et al. [15]	Spain	2021	72	Female	Paroxysmal atrial fibrillation (CHA₂DS₂-VASc score of 4), major gastrointestinal bleeding	Watchman	Fever, chills, and left arm pain (venous catheter)	Not mentioned	IV cefazolin
Aguilera J et al. [16]	United States	2021	73	Male	Atrial fibrillation, coronary artery disease	Watchman	Fever, chest pain, and confusion	Not mentioned	Not mentioned
Chaus A et al. [17]	United States	2020	88	Female	Paroxysmal atrial fibrillation, CHA₂DS₂-VASc score of 5, HAS-BLED score of 3, gastrointestinal bleeding	21 mm Watchman	Presyncope	Not mentioned	Not mentioned
Al Jayyousi B et al. [18]	United States	2022	85	Female	Paroxysmal atrial fibrillation, sick sinus syndrome, dual-chamber pacemaker	Watchman	Fever, weakness, cough, and upper back pain	Not mentioned	Not mentioned
Sam RM et al. [19]	United States	2024	68	Male	Atrial fibrillation	Watchman	Not mentioned	Not mentioned	Not mentioned
Sachdeva A et al. [20]	United States	2023	68	Male	Alcoholic liver cirrhosis, atrial fibrillation, ischemic cardiomyopathy, ventricular tachycardia, sick sinus syndrome, and multiple implantable cardiac devices	Watchman	Sepsis, decompensated heart failure	Not mentioned	Not mentioned
Our case	United States	2025	84	Male	Paroxysmal atrial fibrillation, CHA₂DS₂-VASc score of 5, sick sinus syndrome with dual-chamber pacemaker, chronic diastolic heart failure	Watchman	Fever, chills, and rigors	Cardiac arrest, cardiac tamponade	None

The onset of infection, blood culture results, size of the mass, and medical and surgical therapies are mentioned in Table 2. The mean time to infection following placement of the LAAO device was 10.6 months, and the median time was three months among 13 cases. In one case, the time was mentioned as “several weeks,” and in one case, it was not mentioned. The time ranged from 0.2 months to 36 months. The diagnostic modality of choice was transesophageal echocardiography, which was conducted in all cases and helped localize the lesion and determine its size on the infected device. FDG PET/CT was performed in one case to determine FDG uptake and assess infection of the LAAO device.

**Table 2:** Time of onset of infection, blood cultures, size of the mass, medical and surgical therapy, systemic embolization, outcomes, and follow-up. CIED: Cardiac Implantable Electronic Device; CoNS: Coagulase-Negative Staphylococci; FDG: Fluorodeoxyglucose; FDG PET/CT: Fluorodeoxyglucose Positron Emission Tomography/Computed Tomography; IE: Infective Endocarditis; LAAO: Left Atrial Appendage Occlusion; MRSA: Methicillin-Resistant Staphylococcus aureus; TEE: Transesophageal Echocardiography.

Study / Case	Time between procedure and development of IE	Blood Culture Organism	Invasive procedures	Likely source of infection	Vegetation Size and Location	Medical Therapy	Surgical Removal	Embolization	Outcome	Follow-up
Khumri TM et al. [8]	6 days	Staphylococcus aureus	Pericardiocentesis for a large pericardial effusion due to pericarditis, 2 days after LAAO insertion	Lack of sterile conditions during implantation	5 mm-thick echodensity along the atrial aspect of the LAAO device, extending into the mitral annulus	Initially vancomycin, then switched to nafcillin	Yes	No	Discharged home	Alive at 6 months
Boukobza M et al. [9]	30 months	Pseudomonas aeruginosa	None	Recent blood transfusions	Large plurilocular formation fixed to the LAAO device	Cefotaxime + ciprofloxacin + amikacin	Yes	Yes	Died from refractory cardiogenic shock	-
Jensen J et al. [10]	13 weeks	*Enterobacter *and *Enterococcus*	None	Diverticulitis	1.38 × 1.58 cm echodensity attached to the Watchman device with some mobile components	Not known	Yes	No	Discharged home after a prolonged postprocedural course	Alive at 10 months
Madanat L et al. [11]	12 months	Staphylococcus aureus	Right subclavian central venous catheter	Central venous catheter	1.35 × 0.45 cm mass attached to the device	Initially vancomycin, then piperacillin-tazobactam, later switched to cefazolin	No	No	Discharged home	Alive at 6 months
von Roeder M et al. [12]	3 years	Staphylococcus aureus	Not mentioned	Unknown	Gelatinous mass with floating parts on the LAAO device	Vancomycin	Yes	Yes	Discharged home	Not mentioned
Al-Terki H et al. [6]	20 months	Staphylococcus aureus	None	Unknown	0.9 × 0.8 cm mass located on the LAAO device	Initially metronidazole and ceftriaxone, then switched to flucloxacillin	No	No	Died in the palliative unit	-
Lazarus JJ et al. [13]	24 months	Methicillin-sensitive coagulase-negative *Staphylococcus* (CoNS)	None	Unknown	LAAO vegetation size not known; TEE showed a 10 × 5 mm nonmobile echodensity on the ventricular aspect of the right cusp of the aortic prosthesis. FDG PET/CT showed uptake surrounding the entire LAAO device	Broad-spectrum antibiotics	Yes	No	Discharged home	Not mentioned
Joury A et al. [14]	10 days	MRSA	None	Diabetic foot infection	3 × 2 cm well-organized echodensity with an echolucent rim surrounding an echodense center adherent to the surface of the device	Ceftaroline and daptomycin	No	No	Not mentioned	Not mentioned
Jiménez Martínez C et al. [15]	12 days	MRSA from venous catheter tip	None	Midline venous catheter	Pedunculated mobile mass (1.5 × 1.7 cm) attached to the LAAO device	Ceftaroline and daptomycin	Yes	No	Discharged	Not mentioned
Aguilera J et al. [16]	3 weeks	Serratia marcescens	None	Unknown	Large mobile vegetation on the LAAO device	Not known	No	Septic embolism involving the coronary artery, brain, and intestines; treated medically with lifelong suppressive therapy	Discharged	Not mentioned
Chaus A et al. [17]	1 month	Staphylococcus epidermidis	None	Unknown	Echodensity consistent with vegetation on the LAAO device; echodensities also present on the aortic valve and posterior leaflet of the mitral valve	Not known	No	Not known	Died due to septic shock during hospitalization	-
Al Jayyousi B et al. [18]	10 months	Methicillin-sensitive *Staphylococcus aureus*	None	Unknown	Large echodensity (1.3 × 1.9 cm)	Gentamicin + cefazolin, then switched to cefazolin for 6 weeks	Yes	No	Discharged	Not mentioned
Sam RM et al. [19]	Several weeks	Methicillin-sensitive *Staphylococcus aureus*	Septic arthritis of the right hip requiring incision and drainage	Joint infection	Mobile cystic mass attached to the surface of the Watchman device	Not known	Yes	No	Discharged	Full recovery with IV antibiotics; duration not mentioned
Sachdeva A et al. [20]	Not mentioned	Methicillin-sensitive *Staphylococcus aureus*	Not mentioned	Unknown	Hyperechoic mobile density measuring 1 cm attached to the anterior aspect of the Watchman device	IV cefazolin and oral rifampicin for 6 weeks	No	No	Discharged; vegetation completely resolved on repeat TEE	Not mentioned
Our case	2 weeks	Coagulase-negative *Staphylococcus*	None	Unknown	1.4 × 1.2 cm mobile echodensity attached to the Watchman device; no peri-device leak	Vancomycin and oral cefuroxime for 6 weeks	Yes	No	Discharged to long-term care	Died at 6 months

Five patients had concomitant infections. Four patients had concurrent valvular endocarditis: two patients had mitral valve involvement [9,12], one patient had aortic valve involvement [13], and one patient had both aortic and mitral valve involvement [17]. One patient had a concurrent CRT-D lead infection [12]. Organisms identified from blood cultures suggested a predominance of gram-positive infections, with gram-positive organisms identified in 12/15 patients (80%) and gram-negative organisms in 3/15 patients (20%). Among the gram-positive organisms, the predominant organism was *Staphylococcus aureus*, with six patients having methicillin-sensitive *Staphylococcus aureus* (MSSA), three patients having MRSA, and three patients having coagulase-negative *Staphylococcus*, including one patient in whom the organism was specifically identified as *Staphylococcus epidermidis*. Gram-negative organisms included *Pseudomonas*, *Serratia marcescens*, and *Enterobacter* with *Enterococcus*.

Three patients had undergone invasive procedures, and for two patients, the invasive procedures, namely right subclavian central venous catheter placement in one patient and incision and drainage of septic arthritis in the other patient, were considered potential sources of infection. The third patient underwent pericardiocentesis, but a lack of sterile conditions during implantation was considered a potential source of infection.

Clinical manifestations predominantly consisted of fever, seen in 9/15 patients (60%), followed by signs of sepsis in five patients (33.33%), chest pain in 2/15 patients (13.33%), presyncope in 2/15 patients (13.33%), altered mental status in 2/15 patients (13.33%), a mechanical fall in one patient likely due to diabetic foot infection, and decompensated heart failure in one patient. Clinical findings were not mentioned in one patient. Patients with MRSA infection were treated with vancomycin in one patient and ceftaroline with daptomycin in the other two patients. Patients with MSSA were treated with nafcillin, cefazolin with or without oral rifampicin, or flucloxacillin. Pseudomonas infection was treated with amikacin, cefotaxime, and ciprofloxacin. Our patient with coagulase-negative *Staphylococcus* infection was treated with oral cefuroxime and intravenous vancomycin for six weeks.

Nine patients underwent surgical excision of the LAAO device. The size of the mass attached to the LAAO device ranged from 0.5 cm to 3 × 2 cm. Septic embolization was present in three patients (20%), with cerebral septic embolization in three patients and splenic, intestinal, coronary, and retinal embolism in one patient (2 Senner). One patient who had septic embolization and did not undergo surgical removal of the device ultimately required lifelong suppressive antibiotics [16]. Mortality was observed in 4 of 15 patients (26.66%), and three patients died during hospitalization. All patients who died were >80 years of age and had multiple comorbidities; two had undergone surgical removal of the device, and two had not. Our patient was discharged to long-term care and completed six weeks of antibiotics but eventually died due to severe hypoxia associated with pleural effusions.

Infections related to cardiac devices are rare but are among the most dreaded complications, with substantial healthcare costs [21-26]. Data on the incidence and management of LAAO device infections are lacking. The early postoperative period is the time of highest risk for device infection; therefore, existing preventive strategies concentrate mainly on reducing the risk during that period [27,28]. These infections typically manifest either as pocket-related or systemic infections. Nonspecific symptoms and an unpredictable and usually prolonged interval between microbial seeding and the onset of symptoms further complicate the clinical scenario. Diagnosing a systemic infection is challenging without obvious evidence of local inflammation [29]. In some instances, late device infections can occur and may arise from non-cardiac sources such as respiratory, urogenital, gastrointestinal, or orthopedic infections [30]. According to the 2019 International CIED Infection Criteria [31], second- and, if necessary, third-level diagnostic imaging is recommended when a positive blood culture is found, independently of clinical signs of pocket infection. Subsequently, patients may need to be referred to a higher-level center with expertise in managing CIED infection and extraction.

Timely diagnosis of cardiac device-related infections is of utmost importance, as device extraction is important for complete cure rather than antibiotic therapy alone. A meta-analysis of 11 studies found that device extraction was associated with significantly lower mortality (OR 0.22, 95% CI 0.09-0.50, I² = 62.8%) compared with antibiotic therapy alone [32]. A delay in device extraction (>7 days after diagnosis) was associated with significantly increased in-hospital mortality.

In the nationally representative, all-payer Nationwide Readmissions Database, Lee JZ et al. analyzed patients undergoing transvenous lead removal for device infection [33]. Among 12,999 patients who underwent lead removal for device infection, 8,834 (68%) underwent early removal (≤7 days), and 4,165 (32%) underwent delayed lead removal (>7 days). Delayed lead removal was associated with a significant increase in in-hospital mortality (8.3% vs. 3.5%; adjusted OR 1.70; 95% CI 1.43-2.03; P < 0.001). This finding is further supported by US data from Sciria CT et al., who showed that only 11.5% of the 25,000 CIED patients admitted with endocarditis between 2016 and 2019 were managed with device extraction, with significantly higher mortality among those without extraction (9.5% vs. 6.0%, P < 0.001) [34]. Also, in a more recent study by Pokorney SD et al., among 11,304 patients with CIED infection, device extraction was performed within 30 days in only 18.6% of cases [35].

In this study, we present a rare case of Watchman device infection in an 84-year-old patient. Management included surgical removal of the Watchman device along with six weeks of antibiotic therapy with vancomycin and cefuroxime. The patient was discharged to a rehabilitation facility but eventually passed away within six months due to severe hypoxia and bilateral pleural effusions. We also conducted a systematic review of LAAO device infections and identified 14 published cases. The development of device-related IE consists of multiple independent events. The index event is believed to be microorganisms adhering to pre-existing non-bacterial thrombotic material attached to indwelling prosthetic electrodes or devices [6]. Hence, during a phase of transient or persistent bacteremia, microorganisms can colonize the thrombotic material on intracardiac prosthetic material. It is further postulated that incomplete sealing of the LAA ostium is associated with thrombus formation, as in the majority of cases, the thrombus was detected between the device and the uncovered portion of the limbus because of suboptimal device sizing [36].

There are no studies demonstrating the prevalence of infections in LAAO devices. The EWOLUTION study reported only one case of infection among 1,021 patients who received a Watchman device, and the infection was not well described [37]. There are no clear diagnostic criteria specifically for IE related to intracardiac devices. The diagnosis depends on clinical and laboratory signs of infection, positive blood cultures, and imaging with echocardiography. In many cases, FDG PET scanning, MRI, and soft-tissue ultrasound can be helpful when the above tests are inconclusive. The meta-analysis by Juneau D et al. showed a sensitivity of 87% and specificity of 95% for FDG PET scanning in diagnosing CIED infections [38]. A case report showed incidental FDG uptake at the AtriClip device on PET scanning, which was originally performed for surveillance of pulmonary nodules, in the absence of infection-related signs and symptoms [39]. In our review, the diagnostic modality of choice was TEE, which showed masses/echodensities attached to the Watchman device in almost all patients; however, in one patient, TEE showed no echodensity on the device, and an FDG PET scan was performed, which showed increased uptake surrounding the entire LAAO device. Hence, we suggest that all patients with suspected LAAO device infections undergo TEE, and if the diagnosis remains uncertain, FDG PET scanning should be considered. If an FDG PET scan is performed for other indications and shows incidental uptake around the device, close monitoring of the patient should be undertaken with blood cultures, TEE, and clinical follow-up for signs of infection or sepsis.

In our review, the mean time to infection following placement of the LAAO device was 10.6 months, and the median time was three months. Infection was predominantly caused by gram-positive organisms, with *Staphylococcus aureus* being the most common and a substantial proportion of patients having MRSA. Hence, initial empiric therapy with vancomycin and gram-negative coverage with ceftriaxone, similar to the management of other CIED infections, may be reasonable depending on the clinical context. If patients are unable to receive vancomycin, ceftaroline and daptomycin may be considered depending on organism susceptibility and clinical circumstances. If there is clinical suspicion of Pseudomonas infection, piperacillin-tazobactam may be considered. Antibiotics can eventually be narrowed according to culture and susceptibility results. The key management of infection would be surgical removal of the device, similar to other CIED-related IE [7]. However, many patients are poor surgical candidates for device removal.

In our review, a total of six patients were treated without surgical removal of the device; one patient was alive at six months post-discharge, two patients died in the hospital, and three patients were discharged, but long-term survival and follow-up were not reported. For such patients, an extended duration of antibiotics may need to be considered, often for at least six weeks depending on the organism, clinical course, and source control. The case report by Sachdeva A et al. mentions complete resolution of the vegetation on TEE after six weeks of antibiotic therapy [20]. Septic embolization was present in 20% of patients, with involvement of the brain, spleen, intestine, coronary arteries, and retina. In general, the mortality rate is high in patients with intracardiac device infections, at 13.7% according to the meta-analysis by Ngiam JN et al. [32]. In our review, the mortality rate associated with LAAO device infection was 26.66%. Due to the small sample size and potential publication bias, mortality may be under- or over-reported. Based on the limited data available, surgical removal of the device and source control remain the mainstays of treatment, while patients who are not candidates for surgery require aggressive management with antibiotics. Antimicrobial management should be individualized according to established IE and CIED infection guidelines, with treatment guided by organism identification, antimicrobial susceptibility testing, and patient-specific clinical factors. The regimens reported in this review reflect the management of individual cases and should not be interpreted as evidence supporting a preferred empiric regimen or treatment duration.

## Conclusions

LAAO device infection potentially carries a high risk of mortality and systemic embolization. The most common organism identified was *Staphylococcus aureus*. Diagnosis is primarily made by TEE, with additional imaging modalities such as FDG PET used when needed. The mainstay of treatment is surgical removal of the device, with aggressive antibiotic therapy for patients who are unfit for surgery. The available evidence is very limited, with only 14 previously reported cases in the literature in addition to the present case. Further multicenter studies and standardized reporting are needed to better characterize the clinical course and develop evidence-based management strategies.
